# Independence in Daily Activities after Stroke among Occupational Therapy Patients and Its Relationship with Unilateral Neglect

**DOI:** 10.3390/ijerph18147537

**Published:** 2021-07-15

**Authors:** Iván De-Rosende-Celeiro, Alba Rey-Villamayor, Isabel Francisco-de-Miguel, Adriana Ávila-Álvarez

**Affiliations:** 1 Occupational Therapy Research Unit in Non-Pharmacological Interventions, University of A Coruña, 15071 A Coruña, Spain; adriana.avila.alvarez@udc.es; 2 Private Practice of Occupational Therapy, 36212 Vigo, Spain; alba.reyv@udc.es; 3 Rehabilitation Service, A Coruña University Hospital Complex, 15006 A Coruña, Spain; isabel.francisco@udc.es

**Keywords:** activities of daily living, anosognosia, occupational therapy, stroke, unilateral neglect

## Abstract

More research is needed to better understand the impact of occupational therapy (OT) in stroke patients and syndromes of unilateral neglect (UN) and anosognosia. A prospective, observational, longitudinal design was conducted on a sample of 27 OT patients. The objectives were to examine: (1) the presence of UN and anosognosia; (2) the functional outcomes; and (3) the association of UN at baseline with functional status at discharge from OT. The outcomes were Barthel (functional independence) and the Rivermead Mobility Index (RMI). The baseline proportion of participants with UN was 33% according to the Star Cancellation Test (STC), and 48.1% according to the Catherine Bergego Scale (CBS) therapist-version. There was a significant difference between the therapist and participant-rated CBS scores (*p* = 0.004). Functional independence improved significantly between the initial and final assessments (*p* < 0.001); the effect size (*r*) was large (*r* = 0.61). There was a significant improvement in RMI scores (*p* < 0.001), which was large in size (*r* = 0.59). Both the STC and CBS-therapist scores were significantly correlated with the Barthel (*p* < 0.001, *p* = 0.005, respectively) and with the RMI (*p* = 0.004, *p* = 0.028, respectively). The participants substantially enhanced their functional status skills. UN and anosognosia were common problems, and neglect was associated with worse OT program outcomes.

## 1. Introduction

Stroke is a common health condition in the adult population [1]. This condition can lead to unilateral neglect (UN) and anosognosia. Both neurocognitive syndromes often occur in parallel in the patient and are particularly common among subjects with non-dominant hemisphere damage, which is often the right cerebral hemisphere [2,3,4]. UN, also referred to as visual–spatial neglect, hemispatial neglect, and visuospatial inattention, is a multifaceted disorder of spatial cognition. People with UN typically fail to attend to, perceive, act upon, or move toward novel or meaningful stimuli presented on the side opposite to a brain lesion, and this failure cannot be explained by primary sensory or motor defects [5,6]. It has been attributed to attentional deficits [2,6,7]. In the acute stage, frequencies of up to 80% have been reported [6]. UN can spontaneously recover in whole or in part within the first months after a stroke, although in many cases the effects are long-lasting [7]. This syndrome may adversely affect patients’ ability to perform many daily living activities and limit the ability to move through the spaces of their daily environment [8,9]. On the other hand, anosognosia refers to a lack of insight/awareness or the underestimation of impairments (e.g., hemiparesis, hemianopia, and UN) due to a brain lesion [10]. It has been associated with inferior parietal and superior temporal brain damage [11]. The literature shows that its frequency varies widely, between 20% and almost 45% [3]. Importantly, this syndrome can result in poorer rehabilitation outcomes because patients may not recognize the need to actively participate in prescribed treatments, judging that they are not necessary [3].

Occupational therapy (OT) interventions can include treatment planning to address UN and anosognosia. OT is a critical component of multidisciplinary rehabilitation programs. OT intervention improves key domains in daily life, such as basic activities of daily living (ADL) and functional mobility. After a stroke, the overall goal of OT is to enable people to reach their optimal potential for independent and participatory living [12]. Several systematic reviews have examined empirical research on the efficacy of the OT programs for adults with stroke. A 2017 review included nine studies conducted with samples of individuals with problems in daily activities after stroke [13]. This review concluded that findings must be interpreted cautiously due to the scarcity of localized studies and the low quality of literature on this topic. Similarly, a 2015 systematic review of OT interventions for adults with stroke has revealed that most of the available evidence appears to support the strategies on functional independence in ADL, although several of the analyzed studies were preliminary in nature or had methodological limitations [14]. Furthermore, the evidence is very limited in relation to other domains of functioning, such as daily mobility [13,14].

Based on the abovementioned findings, more research is needed to better understand the impact of OT services on functional independence in adults with stroke, and little is known about the role of this type of intervention on daily mobility skills. Additionally, UN and anosognosia have important implications for the practice of OT. Previous research on these syndromes offers very variable estimates, and information is limited by the small number of reports and the inclusion of samples with different severities and settings. The objectives of this prospective study, therefore, were: (1) to explore the presence of UN and anosognosia of UN in OT patients with stroke; (2) to determine the functional status outcomes (independence in ADL and daily mobility) in this cohort of individuals; and (3) to examine the longitudinal association of the presence of UN, measured with paper-and-pencil and behavioral procedures at OT admission, with the functional status at the time of discharge from this neurorehabilitation program.

## 2. Materials and Methods

### 2.1. Study Design

The design of this exploratory study was longitudinal, observational and prospective.

### 2.2. Setting and Participants

The research was carried out at the Rehabilitation Service of the A Coruña University Hospital Complex in the Spanish city of A Coruña. This hospital service belongs to the public healthcare system and covers a population of over 550,000 individuals in northwestern Spain. It provides comprehensive rehabilitation for inpatient and outpatient adults, adapted on a case-by-case basis, through a multidisciplinary team. A consecutive sampling method over a 5-month period in 2018 was used. All those with stroke consecutively admitted to the OT ward during the study period were invited to participate in the research. Patients were included when they met the following criteria: (a) 18 years of age or older; (b) admission for treatment of OT following a unilateral hemispheric stroke as diagnosed by neurological examination and neuroimaging; (c) this was their first stroke (in order to avoid interference from previous lesions); (d) a post-stroke interval less than six months; (e) medically stable; (f) normal or corrected-to-normal vision and hearing; (g) having enough mental and physical abilities to understand and follow the assessment procedures, according to the clinical judgment of the rehabilitation physician of the service; and (h) a disability in ADL, which refers to the dependence on personal assistance to perform at least one of the daily activities analyzed in the Barthel Index (BI) [15]. Patients were excluded if the anamnesis revealed evidence of a history of other neurologic or psychiatric conditions, which precluded participation in this study, such as drug/alcohol problems and dementia.

The primary aim of the OT intervention analyzed was to optimize the highest possible level of functional independence in daily life through a multicomponent approach. The intervention included activities and techniques in the following domains: (a) sensorimotor and cognitive skills; (b) functional independence in ADL; (c) training with assistive devices; and (d) advice on housing adaptation [13]. Regarding the treatment to specifically address UN, the OT intervention was characterized by a multidimensional approach covering treatments in a wide variety of categories: (1) visual scanning, which consisted of training to initiate a visual scanning pattern starting from the contralesional space; (2) sustained attention tasks; (3) visuomotor imagery training; (4) activation of the contralesional upper limb, through spontaneous and passive movements in the contralesional space; and (5) trunk rotation strategies. These treatment options were described in the work of Chen et al. (2018) [16], based on various stroke rehabilitation guidelines. All participants received an individualized combination of all types of OT domains, adapted on a case-by-case basis. The OT sessions lasted about 30–45 min and were typically conducted two to five times per week, as determined by the therapist. An occupational therapist of the multidisciplinary team with extensive experience in the field of neurorehabilitation conducted this intervention.

### 2.3. Ethical Statement

Prior to commencing this research, written ethical approval for this study was granted by the regional ethics committee (Research Ethics Committee of Coruña-Ferrol, identification code 2018/106). All eligible participants received full verbal and written information about the research and signed an informed consent form before entering the study. If the person had severe deficits in communication, the informal caregiver was responsible for providing written informed consent. Data were stored in a secure manner to protect confidentiality. Confidentiality was preserved in accordance with the European Union General Data Protection Regulation 2016/679. The study followed the tenets of the Declaration of Helsinki [17].

### 2.4. Data Collection and Instruments

Participants were assessed on admission to the OT program (baseline assessment = T1) and after completion of this intervention (assessment at discharge from OT = T2). Data were collected by a trained member of the research team. In countries such as Spain, patients can experience long delays in the onset of OT due to a lack of capacity in rehabilitation services [1]. Therefore, in order to achieve a homogeneous sample and focus the analysis of results on the findings obtained in the first months after stroke, in the present study, the T2 assessment was only performed in those patients who had completed the OT intervention within the first six months of the stroke event.

Demographic data were recorded: age, gender, marital status, educational level (according to the international standard classification of the United Nations Educational, Scientific and Cultural Organization [18]), and living alone (yes/no). Clinical data of stroke were extracted from the medical records: type, hemisphere, time to the OT program (days), and impairments (dysarthria, aphasia and hemianopia). Cognitive functioning was analyzed using the Spanish-language version of the Short Portable Mental Status Questionnaire (SPMSQ). Cognitive impairment is indicated with a score >3 points [19]. Regarding the use of the upper limb, the researcher assessed the use of the affected arm during two functional activities, described in the Motor Activity Log instrument [20]: pick up glass, and grasp fork/spoon. Each activity was assessed dichotomously (the ability to use the affected arm for that activity was as good as before the stroke vs. not). The use of assistive devices for mobility was assessed at baseline: wheelchair (only) and the use of walking aids. 

#### 2.4.1. UN and Anosognosia

The assessment tests for UN were classified into the following types: paper-and-pencil (cancellation test) and behavioral procedures.

*Cancellation tests* are the most commonly used paper-and-pencil procedures to identify visuospatial UN [21,22,23]. The Behavioral Inattention Test [24] was developed specifically to measure visuospatial UN in reaching space. In this study, we used a subtest of this validated battery: the Star Cancellation Test (STC). It has been recognized as having the highest individual sensitivity for identifying the presence of UN out of the entire test battery [25]. This cancellation test was performed on a separate sheet of A4 white paper. The STC requires the individual to scan a page of various images and cross out the smaller target stars in an array of 75 distractors (larger stars and other items). The maximum score is 54 (27 small stars placed on each side of the midline). A score of less than 52 was taken to indicate UN (or ≥3 missed stars). No time limit was given. The participants were in a quiet environment, seated in a chair, and the sheet of paper was presented facing the participant’s midline, at a viewing distance of approximately 45 cm. Subjects were not allowed to relocate the stimulus sheet. Moreover, the participants were asked not to move their trunk during the test. The STC is a valid and highly reliable test of UN [26]. It has been shown to be one of the most sensitive tests in many studies and is associated with the performance of daily activities [25,27,28].

*Behavioral scale*. The second method used to identify participants with UN was a standardized behavioral assessment instrument: the Catherine Bergego Scale (CBS) [29,30]. The scoring of the CBS is based upon direct observations of the patient’s functional performance in daily life by a trained occupational therapist. It is a functional checklist consisting of 10 items related to UN in real-life situations (e.g., eating, dressing, grooming, and locomotion). The CBS assesses personal, peripersonal, and extrapersonal negligence [31]. The severity of UN is rated from 0 (no UN) to 3 (severe UN) points in each item. The total score ranged from 0 to 30. Luukkainen-Markkula et al. (2011) [32] defined the presence of UN by a minimum score of 5 on the CBS scale. The CBS is valid, reliable and sensitive to change during rehabilitation [29,30,31]. This behavioral assessment of UN in daily life was found to be more sensitive than conventional paper-and-pencil tasks in detecting UN [21,30,33].

*Anosognosia of UN*. The CBS has a parallel self-assessment questionnaire aimed at detecting the presence of anosognosia of UN. Awareness of UN was evaluated by questioning the participant about their difficulties on the items of the CBS. It uses the same four-point scale as the CBS applied by therapist observations. Anosognosia can be analyzed by comparing the score on the scale (direct observation by a therapist) with the patient’s self-evaluation. The anosognosia score was defined as the difference between the two scores [29].

#### 2.4.2. Functional Status Outcomes

Functional outcome measures were classified into the following domains of daily life: functional independence in ADL and daily mobility. 

*Functional independence in ADL* was measured by the original version of the BI [15]. It is one of the most widely used rating scales for measurements of the degree of personal assistance required to complete 10 basic activities. The BI is an ordinal clinician-rated scale. The 10 items are summed, and the total score ranges from 0 (total dependence) to 100 (total independence). A score of 0–20 indicates total dependence, 21–60 severe dependence, 61–90 moderate dependence, and 91–99 slight dependence [34]. This instrument is the most widely used to evaluate ADL in studies on post-acute rehabilitation interventions in the population of individuals with stroke [35].

*Daily mobility* was assessed using the Rivermead Mobility Index (RMI) [36,37]. It is a scale of mobility disability that concentrates on body mobility. This instrument was developed in 1991 for patients who had suffered a stroke or head injury. The RMI is a simple measure that consists of 15 items: questions about 14 activities (self-reported data) and one direct observation. It covers a range of activities. Some examples are lying to sitting, transfers, walking inside, walking outside on uneven ground, and bathing. The sum range is 0–15. Zero means that the examined person is not able to perform any of the tested activities correctly. A high total score indicates better mobility performance. 

### 2.5. Data Analysis

Descriptive statistics were used to summarize the results. The categorical variables were reported as frequencies and percentages. The Shapiro–Wilk test was used to determine the normal distribution. The variables that followed a normal distribution were described using the mean and the standard deviation (SD); those that did not follow the normal distribution and the ordinal variables, were described using the median and the first and third quartiles (Q1–Q3). 

We applied the Spearman’s rank correlation to determine the association between the cancellation (STC) and behavioral (CBS) instruments. The strengths of the correlations increase, from 0 to +1, and 0 to −1. The sign shows the direction of the relationship. We interpreted the correlation coefficients using the criteria of Dancey and Reidy (2007) [38]: a value of 0.10 constitutes a weak relationship, 0.40 a moderate relationship, and 0.70 a strong relationship. The Wilcoxon’s signed-rank nonparametric test was used to determine whether there was a significant difference between the assessor and participant CBS scores.

Regarding the BI and RMI instruments, the changes in the scores between the T1 and T2 assessments were tested for significance by means of the Wilcoxon’s signed-rank test. The effect size (*r*) (ES(*r*)) was calculated through the method of Cohen (1988) [39]. An ES(*r*) of 0.10 constitutes a small effect, 0.30 a medium effect, and 0.50 a large effect [39]. The Spearman’s *rho* was also used to evaluate the relationship between the presence of UN at baseline (STC and CBS scores) and the total score for each of the outcome variables of the OT intervention. The IBM SPSS 25.0 (IBM Corporation, Armonk, NY, USA) was used for the statistical analysis. Throughout the study, the level of statistical significance was set a priori at a *p*-value of <0.05, and all tests were two-sided.

## 3. Results

During the study period, 27 individuals with stroke were admitted to OT. All met the inclusion criteria, and all gave their informed consent. These 27 individuals served as the participants in this study and completed the first assessment. Table 1 details the information gathered in the initial assessment for the purpose of describing the characteristics of the sample. The median age was 62.8 years. Most of the participants were married men with a primary education level and lived with others. The most common type of stroke was ischemic. The time from stroke to admission for the OT program was 52 days (median). Almost 60% of the participants had dysarthria (59.3%). The median SPMSQ score was 2, representing an absence of cognitive impairment. More than 65% of the participants had no ability to grasp a fork or spoon. More than one-third used a wheelchair for mobility at baseline. Regarding the functional status, the median BI score was 70 points, representing a moderate level of dependence in ADL. In the RMI, the median score was 7 points. 

### 3.1. Objective 1: Presence of UN and Anosognosia

*UN* (*n* = 27). Concerning the assessment of the presence of UN at T1, the median STC score was 53 (range 9–54, Q1–Q3 = 49–54). Nine participants (33.3%) had UN according to the STC scale. In the therapist version of the CBS, the median score was 4 (range 0–17, Q1–Q3 = 2–9), and 13 participants (48.1%) had UN as defined by Luukkainen-Markkula et al. (2011) [32]. There was a significant correlation (Spearman’s ρ = −0.738, *p* < 0.001) between the STC and CBS (therapist) scores. Regarding the group of patients with ischemic stroke (*n* = 24), the percentages of participants with UN according to the STC and the CBS-therapist were 33.3% and 50%, respectively.

*Anosognosia of UN* (*n* = 27). In the self-assessment questionnaire of CBS, the median score was 2 points (range 0–17, Q1–Q3 = 1–6) at baseline, and eight participants (29.6%) had UN. There was a statistically significant difference between the assessor and participant-rated CBS scores. Total scores were significantly higher on the version applied by therapist observation than on the patient-rated scale (*p* = 0.004). Although a significant correlation was found between therapist and patient-rated scores, the strength of the correlation did not reach a value of 0.7 (Spearman’s ρ = 0.629, *p* < 0.001). In addition, no statistically significant correlation was found between the self-rated version of the CBS and the STC (*p* = 0.201). The median anosognosia score was 0 points (Q1–Q3 = 0–4). The anosognosia score was significantly correlated with the severity of UN (Spearman’s ρ = 0.457, *p* =0.017). Similarly, in patients with ischemic stroke (*n* = 24), the median anosognosia score was 1.5 points (Q1–Q3 = 0–4).

### 3.2. Objective 2: Functional Status Outcomes

The T2 assessment was carried out in the 17 patients (63%) who completed the OT program within the first six months from the stroke event. In this group, regarding the weekly frequency of the OT sessions, the participants received a median of three sessions per week (Q1–Q3 = 2.5–5).

#### 3.2.1. Functional Independence in ADL

**Figure 1:**
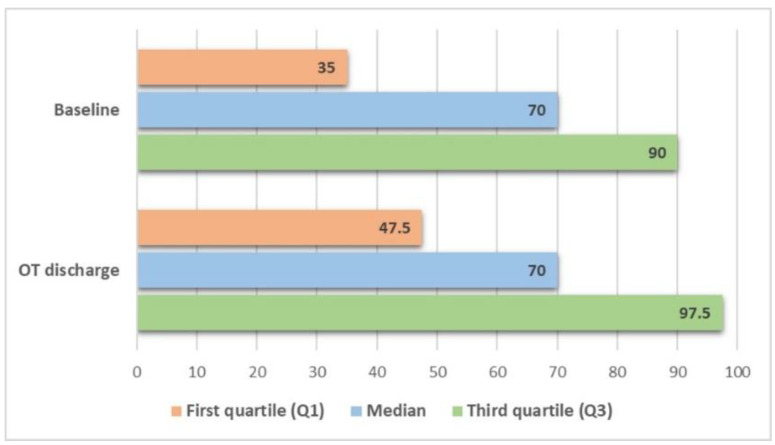
Functional independence, before and after the occupational therapy (OT) program (*n* = 17).

Figure 1 details the changes in BI scores, before and after the OT program (*n* = 17). The Wilcoxon test showed a statistically significant improvement in functional independence in ADL from the T1 to T2 assessments: 16 subjects improved their score and 1 remained unchanged (*p* < 0.001). The ES(*r*) was 0.61. This improvement was also statistically significant in the group of participants with ischemic stroke (*n* = 14, *p* = 0.001).

#### 3.2.2. Daily Mobility

**Figure 2:**
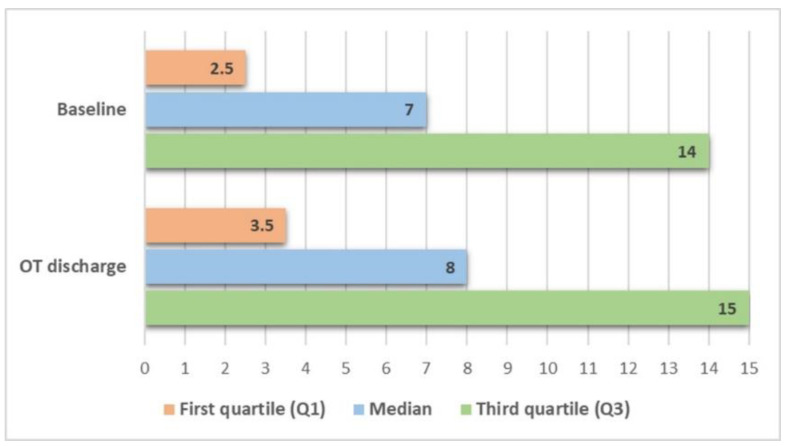
Daily mobility, before and after the occupational therapy (OT) program (*n* = 17).

Figure 2 shows changes in RMI scores between the baseline and T2 assessments (*n* = 17). Statistically significant changes were observed when comparing the total score on this scale before and after the OT program: the median score in the initial evaluation was 7 at baseline, and this total score was 8 (median) at T2 (*p* < 0.001). The ES(*r*) was 0.59. These statistically significant changes were also found in the group of patients with ischemic stroke (*n* = 14, *p* = 0.001).

### 3.3. Objective 3: Relationship between UN and Functional Status

#### 3.3.1. Cancellation Test

**Figure 3:**
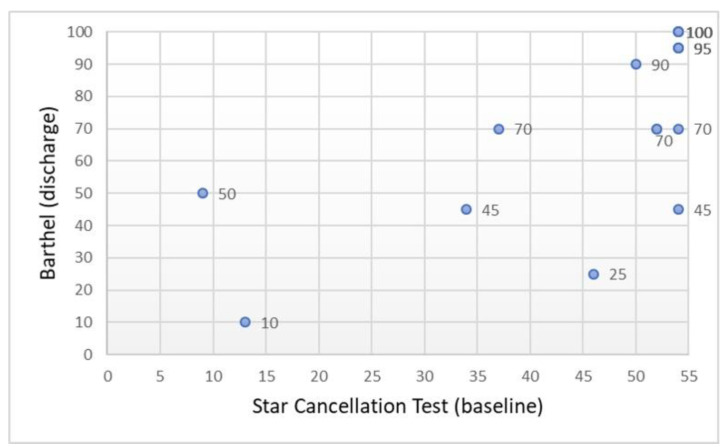
Scatter plot showing the relationship between the cancellation test and functional independence in ADL (*n* = 17).

**Figure 4:**
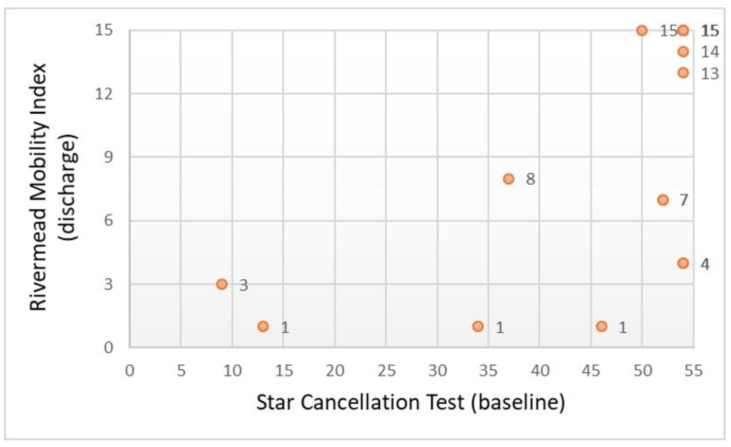
Scatter plot showing the relationship between the cancellation test and daily mobility (*n* = 17).

Table 2 shows the correlations between the cancellation test and the functional status (*n* = 17). The STC scores assessed at admission to OT were significantly correlated with the total BI and RMI scores assessed at OT discharge. There was a positive correlation between STC scores and these two scales of OT functional outcomes (Figure 3 and Figure 4). The strength of the correlation was greater with the BI, exceeding the value of 0.70. Similarly, these correlations were also statistically significant in the group of participants with ischemic stroke (*n* = 14).

#### 3.3.2. Behavioral Scale

**Figure 5:**
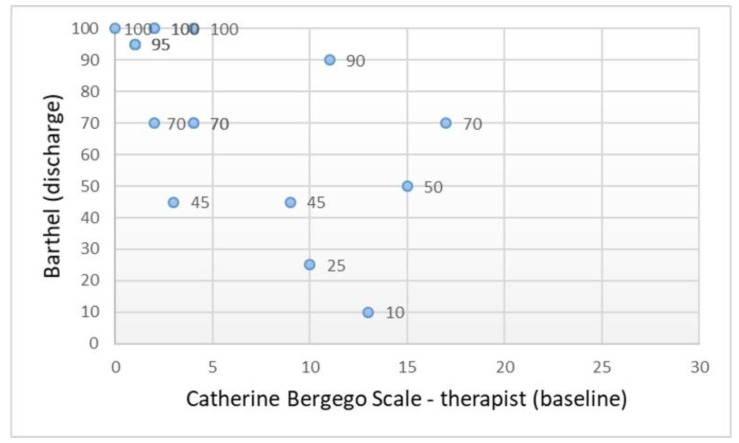
Scatter plot showing the relationship between the behavioral scale and functional independence in ADL (*n* = 17).

**Figure 6:**
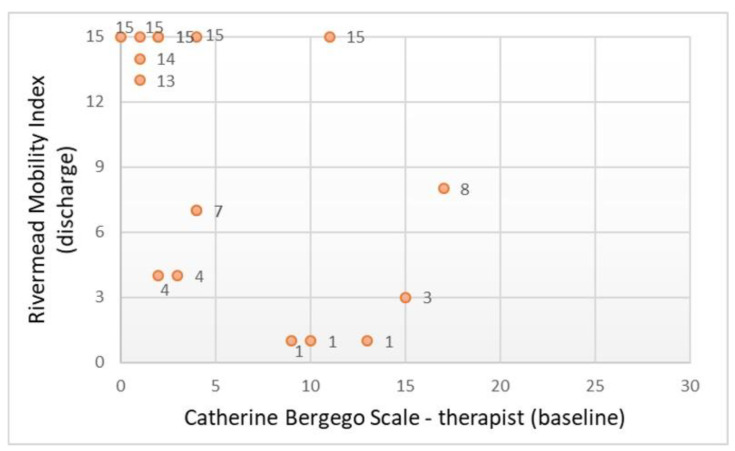
Scatter plot showing the relationship between the behavioral scale and daily mobility (*n* = 17).

The CBS scores assessed by the therapist at T1 were significantly correlated with the total BI and RMI scores assessed at the time of OT discharge (Table 2, *n* = 17). These correlations were negative (Figure 5 and Figure 6) and exceeded a value of 0.5. However, no significant associations were found between the participant-rated CBS and the two instruments of functional status (Table 2). Consistent with these findings, in the group of patients with ischemic stroke (*n* = 14), the correlation between CBS-therapist scores and the two outcome scales was negative and statistically significant. In this ischemic stroke group, no significant associations were found between the CBS-patient and the two instruments of functional status.

## 4. Discussion

Using data from a sample of middle-aged patients, mostly outpatients with unilateral brain damage referred to OT between the first and second month after their first stroke, this research provides detailed information on different areas of relevance to OT practice. Post-OT values on the BI and RMI scales were significantly higher than those observed at baseline. Moreover, to the best of our knowledge, this is the first prospective study exploring the frequency of UN and anosognosia in a Spanish cohort of OT patients. The findings showed that both syndromes were common problems, and UN was associated with worse functional status at the time of OT discharge.

This study extends previous research by examining epidemiological and clinical data on UN and anosognosia among OT patients. The results indicated that between 33% and 48% of the participants had UN, in accordance with the cancellation and functional assessment procedures used, respectively. Although a strong correlation was found between the STC and CBS (therapist) scores, discrepancies were observed between the classical tests and the behavioral instruments, consistent with the findings of previous studies in the literature [30,32,33]. Previous studies coincide in identifying lower frequencies of UN in the paper-and-pencil tests [30,32,33], attributing it to lower sensitivity [30] and limited ecological validity [31] compared to naturalistic observation instruments. It is difficult to compare our results with the incidence rates of previous research due to the existence of considerable methodological differences between studies. A review of 30 studies on the frequency of UN after stroke concluded that it is not possible to establish a reliable estimate of the prevalence from the existing literature, because it is extremely variable due to important differences in the areas of subject selection, lesion localization, and the nature and timing of assessment [40]. It has been observed that the frequency is higher in the first weeks, progressively decreasing over time [40,41]. The high prevalence identified in our study may be related to the selection criteria, because the sample is made up of patients referred to OT, which represents a subgroup of patients with rehabilitation needs derived from poor spontaneous recovery. Another aim of this work was to explore the presence of anosognosia of UN. The patients perceived significantly less negligence than the therapists, reflecting the lack of awareness or the underestimation of this deficit. The difference between the therapist and patient scores was two points (median), similar to the data collected in the recent secondary analysis of two post-stroke rehabilitation studies [42]. This difference was six points in the work of Azouvi et al. (2003) [30], although the authors only included right-hemisphere stroke patients. Another result that points to the existence of anosognosia of UN is the absence of a significant association between the STC instrument and the self-assessment version of the CBS. Finally, considering findings such as the high frequency of UN and anosognosia and the wealth of information provided by the assessment instruments used, we can conclude that occupational therapists should pay special attention to these neurological syndromes in the evaluation processes, through the complementary use of different types of instruments that cover the therapist’s point of view, the objective vision of the traditional tests, and the self-assessment perspective. A careful comprehensive evaluation is essential to establish adequate intervention strategies to lessen the impact of these problems.

The results showed a statistically significant improvement in the domains of independence in ADL and the performance of functional mobility. The effect sizes were large. Our study, therefore, provides an optimistic view of the potential for improvement of the analyzed population. Functional independence was measured as an improvement in the ability to perform ADL. The improvement in ADL found in our research was consistent with the few studies included in the literature in this field [13,14]. However, the comparisons between the results with previous literature are complicated due to differences in the study populations, settings and intervention modalities. In the most recent systematic review focused on this topic, a Cochrane review on OT intervention in the area of daily activities of adults with stroke included only five studies with results based on BI scores [13]. None of these studies was carried out by a rehabilitation hospital service, and most of the samples were characterized by ages close to or greater than 70 years. On the other hand, many OT patients had marked mobility limitations, and more than one-third used a wheelchair at baseline. From a clinical perspective, daily mobility is an important goal for OT practice because it encompasses a set of skills necessary to achieve a return to meaningful activities in the community [12]. However, although RMI has been demonstrated as a valid and reliable for assessing mobility in stroke patients [43,44], no studies were found that systematically assessed OT outcomes on RMI scores in the study population. It is hoped that this study will help to form a basis for future research on the longitudinal effects of OT on daily mobility activities in this population.

The presence of UN after stroke was significantly associated with functional status at OT discharge. People with UN were more likely to complete OT with a lower level of independence in ADL and poor performance in daily mobility. The strength of the relationship ranged between moderate and strong, obtaining higher values in relation to functional independence (BI). The results are consistent with previous studies which have suggested that UN plays an important role as a barrier to restore independent functioning at home and in the community. A few studies have found evidence on the relationship between UN and IB scores, although there is great heterogeneity in the study populations analyzed: in a sample of patients referred to a rehabilitation unit an average of 19 weeks after a right-hemisphere stroke [29], individuals were managed on a stroke unit in the first days after the stroke event [45], and older adults were included from an inpatient rehabilitation unit [46]. In line with our findings, a study of rehabilitation inpatients with a mean age of 69 years showed a significant association between UN and functional mobility, as measured by the MRI instrument [46]. Regarding the clinical implications for practice in neurorehabilitation settings, the results shed light on the need to consider UN when planning OT programs aimed at improving the daily functioning of this population group. This study suggests that special emphasis needs to be placed on the early development of specific interventions aimed at treating this neurocognitive syndrome as a key means of preventing or reducing the functional limitations associated with its presence at the time of admission to OT.

Despite the meaningful findings, we note several limitations in the study design which limit the conclusions that can be drawn from this exploratory research. The reader must keep in mind that the study design did not allow inferences on causal relationships. It should be noted that this research was a prospective observational study, not a randomized controlled trial. The absence of a control group was an important limitation; therefore, the changes that occurred during the study cannot be attributed to the effects of the OT intervention alone. It is not possible to ascertain the influence on the results of other rehabilitation treatments that the participants received, such as physiotherapy. Furthermore, we did not perform a long-term follow-up. Consequently, we cannot be sure that the functional outcomes we observed during the OT program persisted afterward. Another concern is the possible selection bias, because it is the rehabilitation physician who determines access to the study, according to clinical judgment. Additionally, characteristics of the OT intervention, such as the length of the sessions or its contents, were not recorded in our study for practical reasons (limited personnel resources). Finally, the study was conducted at one site only. Therefore, our results cannot be generalized to the overall population of neurorehabilitation stroke patients. The sample size was small, which limits the ability to create predictive models to understand the influence of UN on OT outcomes. These limitations should be specifically addressed by designing multicenter studies, with a larger sample size and a more heterogeneous sample.

## 5. Conclusions

Although this research has its limitations imposed by the design, methodology and sample size, the findings extend the existing evidence base for the OT practice. Regarding the first objective of the study, UN and anosognosia of neglect were very common post-stroke problems among OT patients. The participants showed significant improvements in various domains of daily functioning (functional independence and daily mobility), and the effects on functional status were large (second objective). Finally, the results for the third objective highlight the importance of paying attention to assessments and interventions in the area of UN, due to the presence of a moderate to strong relationship between this neurocognitive syndrome and worse OT program outcomes.

## Figures and Tables

**Table 1:** Characteristics of the study population at initial assessment (*n* = 27).

Baseline Characteristics	Values ^a^
Gender	
Male	19 (70.4)
Age (years)	
Mean (SD)	62.8 (12.9)
Range	44–87
Marital status	
Married	17 (63.0)
Widow/er	4 (14.8)
Single	4 (14.8)
Separated/divorced	2 (7.4)
Educational level	
Primary education	17 (63.0)
Secondary	7 (25.9)
Tertiary	3 (11.1)
Living alone	4 (14.8)
**Stroke**	
Type	
Ischemic	24 (88.9)
Non-ischemic	3 (11.1)
Hemisphere	
Right	15 (55.6)
Left	12 (44.4)
**Neurorehabilitation**	
Days from stroke to OT	
Median (Q1–Q3)	52 (28–94)
Range	9–173
Outpatient	19 (70.4)
Physiotherapy Speech therapy	20 (74.1) 5 (18.5)
**Functional Status**	
ADL	
Barthel Index: median (Q1–Q3)	70 (60–90)
Range	5–95
Daily mobility	
RMI: median (Q1–Q3)	7 (3–13)
Range	0–15
**Impairments**	
Dysarthria	16 (59.3)
Aphasia	7 (25.9)
Hemianopia	7 (25.9)
Mental Function	
SPMSQ: median (Q1–Q3)	2 (1–3)
Upper extremity (affected)	
Pick up glass: no	9 (33.3)
Grasp fork/spoon: no	18 (66.7)
Assistive devices for mobility	
Wheelchair (only)	10 (37)
Walking aid	4 (14.8)

OT, occupational therapy. ADL, basic activities of daily living. RMI, Rivermead Mobility Index. SPMSQ, Short Portable Mental Status Questionnaire. ^a^ Data are presented as *n* (%) unless otherwise stated.

**Table 2:** Correlations between the presence of unilateral negligence and the functional outcomes (*n* = 17).

	ADL: Barthel Index (T2)		Daily Mobility: RMI (T2)	
Unilateral Neglect Test (T1)	Spearman’s ρ	*p*-Value	Spearman’s ρ	*p*-Value
**Paper-And-Pencil Test**				
Star Cancellation Test	0.728	*p* < 0.001 ^a^	0.657	0.004 ^a^
**Behavioral Scale**				
CBS-therapist	−0.650	0.005 ^a^	–0.532	0.028 ^a^
CBS-patient	NS	0.680	NS	0.922

ADL, basic activities of daily living. T1, baseline. T2, after completion the occupational therapy program. RMI, Rivermead Mobility Index. CBS, Catherine Bergego Scale. ^a^ Indicates statistical significance.
